# Pill burden and health-related quality of life across dialysis modalities: a multimodal assessment using KDQOL-36 and eQ-5D-5L

**DOI:** 10.1080/0886022X.2026.2713235

**Published:** 2026-08-06

**Authors:** Suthiya Anumas, Tassamon Jankaew, Montira Khienlikit, Siriphat Simapaisan, Thitikarn Jungteerapanich, Chanon Chiarnpattanodom, Tinpawee Thongkongthun, Thanakhom Hoontrakul, Poomsit Rattanapanop, Pattharawin Pattharanitima

**Affiliations:** a Chulabhorn International College of Medicine, Thammasat University, Pathum Thani, Thailand; b Division of Nephrology, Department of Internal Medicine, Faculty of Medicine, Thammasat University, Pathum Thani, Thailand; c Thammasat University Hospital, Pathum Thani, Thailand; d Faculty of Medicine, Thammasat University, Rangsit, Pathum Thani, Thailand

**Keywords:** Dialysis, EQ-5D-5l, kidney disease quality of life (KDQOL-36), pill burden, quality of life

## Abstract

**Background and hypothesis:**

Patients receiving maintenance dialysis experience substantial pill burden from complex comorbidities and intensive treatment regimens. Although higher pill burden is linked to treatment burden and poorer health-related quality of life (HRQoL), optimized pharmacologic management may improve overall well-being. We evaluated the association between pill burden and HRQoL across dialysis modalities.

**Methods:**

We conducted a cross-sectional study of adult patients receiving maintenance hemodialysis (HD), hemodiafiltration (HDF), or peritoneal dialysis (PD) at a tertiary care center. Pill burden was defined as the total number of oral tablets taken daily and categorized as <10, 10–15, or >15 tablets/day. HRQoL was assessed using validated Thai versions of the Kidney Disease Quality of Life-36 (KDQOL-36) and EQ-5D-5L questionnaires. Univariable and multivariable linear regression analyses evaluated associations between pill burden and HRQoL outcomes after adjustment for confounders.

**Results:**

A total of 156 patients were included: 64 HD, 64 HDF, and 28 PD patients. Median pill burden was 11.5, 10, and 7.5 tablets/day in the HD, HDF, and PD groups, respectively. Antihypertensive and CKD–mineral and bone disorder medications were the main contributors. After adjustment, pill burden was not significantly associated with PCS-12, MCS-12, SPKD, EKD, or EQ-5D-5L-VAS scores. However, patients receiving >15 tablets/day had significantly lower BKD scores, while higher pill burden was independently associated with higher EQ-5D-5L index scores. HDF and PD were consistently associated with better HRQoL than HD.

**Conclusions:**

Pill burden demonstrated a complex relationship with HRQoL. Dialysis modality appeared to exert a stronger influence on HRQoL than pill burden itself.

## Introduction

Patients receiving maintenance dialysis are exposed to a substantial pill burden due to complex comorbidities and intensive treatment regimens [1,2]. The pharmacological management of end-stage kidney disease (ESKD) primarily involves antihypertensive agents, vitamin D supplements and phosphate binders [3,4]. Previous studies have reported a significant median (IQR) pill burden in CKD stage 5D of 122 (61) tablets per week [5]. Variations have also been observed across different modalities, with pill burdens differing between peritoneal dialysis (PD), home hemodialysis (Home-HD), and in-center hemodialysis (HD) [6].

The existing literature suggests that a high pill burden is strongly associated with increased treatment burden, reduced medication adherence, and impaired health-related quality of life (HRQoL) [3,7,8]. While the physical act of managing polypharmacy contributes to treatment burden, optimal pharmacological therapy may paradoxically improve overall outcomes by reducing morbidity and stabilizing symptoms, thereby potentially maintaining or even enhancing HRQoL.

Furthermore, the dialysis modality itself may significantly impact both treatment burden and HRQoL. Patients receiving PD may experience better HRQoL because treatment can be performed at home, allowing greater independence and reducing the need for frequent hospital visits. Within extracorporeal therapies, hemodiafiltration (HDF) has emerged as a superior alternative to conventional hemodialysis (HD) due to its enhanced clearance of middle molecules [9], which may further influence the relationship between clinical morbidity and patient well-being.

Since the ultimate goals of ESKD management are to prolong survival while preserving patients’ HRQoL, functional independence, and overall well-being, HRQoL should be considered an important outcome in patients receiving maintenance dialysis. Different dialysis modalities may require varying degrees of pharmacological treatment to achieve adequate biochemical control, resulting in differences in pill burden. Although intensified therapy may improve laboratory outcomes, the associated increase in pill burden may negatively affect patients’ perceived well-being and HRQoL.

Given these complexities, this study aimed to evaluate the association between pill burden and HRQoL across different dialysis modalities. Using validated patient-reported outcome measures (PROMs), including the Thai versions of the Kidney Disease Quality of Life-36 (KDQOL-36) [10] and EQ-5D-5L [11], and adjusting for key clinical confounders, we sought to determine the independent impact of pill burden on HRQoL among patients receiving maintenance dialysis.

## Material and methods

### Study design and participants

This cross-sectional study was conducted among adult patients receiving maintenance dialysis, including HD, HDF, and PD, at Thammasat University Hospital, a tertiary care teaching hospital located in Pathum Thani, Thailand. Dialysis modality was selected according to the treating physician’s clinical judgment, considering standard medical indications and contraindications. However, modality selection was also influenced by reimbursement eligibility and patients’ financial circumstances. PD was generally preferred for patients covered by Thailand’s Universal Coverage (UC) scheme. HD was provided to UC beneficiaries with contraindications to PD and to patients covered by other reimbursement schemes. HDF required an additional out-of-pocket payment by the patient. Eligible participants were patients aged ≥18 years who had been receiving maintenance dialysis for at least 3 months and were able to complete HRQoL assessments. Patients with acute illness, hospitalization during the study period, severe cognitive impairment, or inability to provide informed consent were excluded.

### Data collection

Baseline demographic and clinical characteristics were collected from medical records and patient interviews. Variables included age, sex, dialysis modality, dialysis vintage, underlying comorbidities, current medications, and laboratory parameters. Medication use was reviewed at the time of enrollment, and pill burden was defined as the total number of oral tablets taken per day. Patients were categorized into three groups according to daily pill burden: <10 tablets/day, 10–15 tablets/day, and >15 tablets/day.

### Assessment of health-related quality of life

HRQoL was assessed using validated Thai versions of KDQOL-36 questionnaire and the EQ-5D-5L instrument. The KDQOL-36 consists of five domains, including the Physical Component Summary (PCS-12), Mental Component Summary (MCS-12), Burden of Kidney Disease (BKD), Symptoms and Problems of Kidney Disease (SPKD), and Effects of Kidney Disease (EKD). Higher scores indicate better HRQoL. Scores for each KDQOL-36 domain range from 0 to 100, with higher scores indicating better HRQoL. The EQ-5D-5L index was calculated using the Thai population value set, with higher index scores representing better perceived health status.

### Statistical analysis

Continuous variables were expressed as mean ± standard deviation (SD) or median with interquartile range (IQR), as appropriate, while categorical variables were presented as frequencies and percentages. Baseline characteristics were compared across pill burden categories using one-way analysis of variance (ANOVA), Kruskal–Wallis test, chi-square test, or Fisher’s exact test, as appropriate.

Univariable linear regression analyses were initially performed to evaluate associations between pill burden categories and HRQoL outcomes. Variables considered clinically relevant or showing statistical significance in univariable analyses were subsequently included in multivariable linear regression models. Adjusted analyses were performed to determine the independent association between pill burden and HRQoL outcomes after controlling for age, sex, dialysis vintage, dialysis modality, underlying comorbidities, and laboratory parameters. Regression coefficients (β) with 95% confidence intervals (CIs) were reported. A two-sided p-value <0.05 was considered statistically significant. All statistical analyses were performed using Stata version 17.0 BE (StataCorp, College Station, TX, USA).

### Ethical considerations

The study protocol was approved by the Human Research Ethics Committee of Thammasat University (Medicine), Thailand (Project No. MTU-EC-IM-0-111/67; Certificate of Approval No. 215/2024). The study was conducted in accordance with the principles of the Declaration of Helsinki, the Belmont Report, CIOMS guidelines, and the International Conference on Harmonization Good Clinical Practice (ICH-GCP). All participants provided written informed consent prior to study enrollment.

## Results

### Baseline characteristics

A total of 156 patients receiving maintenance dialysis were included in the study, comprising 64 patients undergoing HD, 64 receiving HDF, and 28 undergoing PD. The median (IQR) pill burden was 11.5 (10) tablets/day in HD patients, 10 (14.5) tablets/day in HDF patients, and 7.5 (9.5) tablets/day in PD patients. Among medication categories, antihypertensive agents and CKD–mineral and bone disorder (CKD-MBD) medications represented the two most common contributors to pill burden, with median values of 3 (5.5) and 3 (4) tablets/day, respectively.

Patients were categorized according to daily pill burden into <10 tablets/day (*n* = 48), 10–15 tablets/day (*n* = 57), and >15 tablets/day (*n* = 51). Dialysis vintage differed significantly across pill burden groups, with patients receiving >15 tablets/day having a shorter dialysis vintage compared with those receiving fewer medications (*p* = 0.009). Hypertension was more prevalent among patients with higher pill burden categories (72.9%, 91.2%, and 96.1%, respectively; *p* = 0.002). Significant differences were also observed in serum potassium and phosphate levels across pill burden groups (*p* < 0.001 and *p* = 0.045, respectively) (Table 1).

**Table 1.** Baseline characteristics.

Variable	<10 tabs/day (*n* = 48)	10–15 tabs/day (*n* = 57)	>15 tabs/day (*n* = 51)	Total (*n* = 156)	*p*-value
**Demographics**					
Age, years, mean (SD)	68.5 (14.9)	66.2 (14.6)	62.1 (17.3)	65.6 (15.8)	0.17
Dialysis vintage, years, median (p25–p75)	4.75 (2.35–7.45)	4.3 (1.4–6.6)	1.7 (1.1–5.5)	3.9 (1.4–6.3)	**0.009**
Male, *n* (%)	23 (47.9)	34 (59.6)	30 (58.8)	87 (55.8)	0.42
**Comorbidities**					
Diabetes mellitus, *n* (%)	12 (25.0)	7 (12.3)	13 (25.5)	32 (20.5)	0.14
Hypertension, *n* (%)	35 (72.9)	52 (91.2)	49 (96.1)	136 (87.2)	**0.002**
Dyslipidemia, *n* (%)	31 (64.6)	43 (75.4)	37 (72.6)	111 (71.2)	0.48
Cardiovascular disease, *n* (%)	10 (20.8)	19 (33.3)	17 (33.3)	46 (29.5)	0.27
Laboratory parameters					
Hemoglobin, g/dL, mean (SD)	10.19 (1.74)	10.52 (1.50)	10.22 (1.28)	10.32 (1.51)	0.32
Sodium, mmol/L, mean (SD)	136.6 (2.69)	136.2 (3.21)	136.1 (3.81)	136.3 (3.26)	0.86
Potassium, mmol/L, mean (SD)	4.15 (0.64)	4.38 (0.57)	4.66 (0.73)	4.40 (0.68)	**<0.001**
Bicarbonate, mmol/L, mean (SD)	24.7 (2.17)	23.9 (2.41)	24.5 (3.12)	24.3 (2.60)	0.28
Calcium, mg/dL, mean (SD)	8.99 (0.71)	8.80 (0.79)	8.83 (0.65)	8.87 (0.72)	0.38
Phosphate, mg/dL, mean (SD)	4.84 (1.89)	4.22 (1.35)	4.86 (1.37)	4.62 (1.56)	**0.045**
Parathyroid hormone, pg/mL, median (p25–p75)	403.5 (249.9–629)	371 (239–646.5)	534 (289–787)	433.7 (247.9–649)	0.31
Albumin, g/dL, mean (SD)	3.65 (0.40)	3.70 (0.35)	3.80 (0.43)	3.72 (0.39)	0.22
25(OH)-vitamin D, pg/mL, mean (SD)	31.5 (16.1)	29.8 (9.2)	30.3 (7.6)	30.5 (11.3)	0.93
Blood glucose, mg/dL, mean (SD)	135.5 (57.4)	128.8 (52.6)	123.8 (48.4)	129.3 (52.7)	0.36
HbA1c, %, mean (SD)	6.18 (1.21)	5.97 (1.31)	5.92 (1.30)	6.02 (1.27)	0.41
**Medication counts**					
Antihypertensive drugs, median (p25–p75)	1 (0–2)	3 (1–4)	10 (4–14)	3 (1–6.5)	**<0.001**
CKD-MBD drugs, median (p25–p75)	2 (1–3)	4 (2–6)	5 (3–8)	3 (2–6)	**<0.001**
Supplements, median (p25–p75)	0 (0–1)	2 (0–4)	3 (1–4)	1 (0–4)	**<0.001**
Lipid-lowering drugs, median (p25–p75)	1 (0–1)	1 (1–1)	1 (0–1)	1 (0–1)	0.50
Sodium bicarbonate drugs, median (p25–p75)	0 (0–0)	0 (0–2)	2 (0–4)	0 (0–2)	**0.04**
Glucose-lowering drugs, median (p25–p75)	0 (0–0)	0 (0–0)	0 (0–0)	0 (0–0)	0.87
Diuretics, median (p25–p75)	0 (0–0)	0 (0–1)	0 (0–1)	0 (0–1)	0.43
Other drugs, median (p25–p75)	0 (0–1)	0 (0–1)	0 (0–1)	0 (0–1)	0.58
Total drugs, median (p25–p75)	7 (5.5–8)	12 (10–14)	23 (18–28)	12 (9–18)	**<0.001**
**Quality of life & symptom scores**					
PCS-12, median (p25–p75)	31.1 (22.3–43.0)	32.4 (25.9–44.6)	36.0 (23.8–45.3)	32.8 (23.3–44.2)	0.50
MCS-12, median (p25–p75)	41.6 (31.5–53.7)	44.7 (33.2–55.1)	44.3 (33.2–56.8)	43.5 (33.2–55.3)	0.17
BKD score, median (p25–p75)	25 (0–78.1)	31.3 (0–68.8)	18.8 (0–56.3)	25 (0–68.75)	0.41
SPKD score, median (p25–p75)	82.3 (71.9–86.5)	81.3 (75–85.4)	83.3 (75–87.5)	81.3 (72.9–87.5)	**0.008**
EKD score, median (p25–p75)	59.4 (26.6–92.2)	62.5 (34.4–81.3)	53.1 (37.5–78.1)	59.4 (31.3–84.4)	0.40
EQ-5D index, median (p25–p75)	0.74 (0.33–0.88)	0.82 (0.72–0.94)	0.82 (0.71–0.94)	0.80 (0.66–0.93)	**0.001**
EQ-VAS, median (p25–p75)	50.0 (17.5–80.0)	50.0 (25.0–75.0)	60.0 (40.0–80.0)	50.0 (25.0–80.0)	0.35
**Dialysis modality**					0.16
HD, n (%)	22 (45.8)	21 (36.8)	21 (41.2)	64 (41.0)	
HDF, n (%)	14 (29.2)	25 (43.9)	25 (49.0)	64 (41.0)	
PD, n (%)	12 (25.0)	11 (19.3)	5 (9.8)	28 (18.0)	

**Abbreviations**: BKD: Burden of Kidney Disease; CKD-MBD: chronic kidney disease–mineral and bone disorder; EKD: Effects of Kidney Disease; EQ-5D-5L: EuroQol 5-Dimension 5-Level; EQ-VAS: EuroQol Visual Analogue Scale; HD: hemodialysis; HDF: hemodiafiltration; IQR: interquartile range; p25: 25th percentile; p75: 75th percentile; MCS-12: Mental Component Summary; PCS-12: Physical Component Summary; PD: peritoneal dialysis; SD: standard deviation; SPKD: Symptoms and Problems of Kidney Disease.

The number of antihypertensive agents, CKD-MBD medications, supplements, and sodium bicarbonate tablets increased significantly with higher pill burden categories (all *p* < 0.05). The median total number of medications was 7 (IQR 5.5–8), 12 (IQR 10–14), and 23 (IQR 18–28) tablets/day in the <10, 10–15, and >15 tablets/day groups, respectively (*p* < 0.001) (Table 1). The pharmacologic classes of medications included in each category are provided in Supplementary Table S1.

### Pill burden and HRQOL

In univariable linear regression analyses, pill burden was not significantly associated with KDQOL-36 domains, including PCS-12, MCS-12, BKD, SPKD, EKD, or with EQ-5D-5L-VAS scores. However, both the 10–15 tablets/day and >15 tablets/day groups were significantly associated with higher EQ-5D-5L index scores compared with the <10 tablets/day group (β 0.18, 95% CI 0.08–0.29, *p* = 0.001 and β 0.19, 95% CI 0.08–0.30, *p* = 0.001, respectively). Older age (≥65 years) was associated with lower PCS-12, EQ-5D-5L index, and EQ-5D-5L-VAS scores, while cardiovascular disease was associated with lower PCS-12, BKD, and EQ-5D-5L index (Table 2).

**Table 2.** Univariable associations between clinical Variables and HRQoL measures among patients receiving maintenance dialysis.

	PCS-12		MCS-12		BKD		SPKD		EKD		EQ-5D-5L Index		EQ-5D-5L-VAS	
Variable	β coefficient (95% CI)	*p*-value	β coefficient (95% CI)	*p*-value	β coefficient (95% CI)	*p*-value	β coefficient (95% CI)	*p*-value	β coefficient (95% CI)	*p*-value	β coefficient (95% CI)	*p*-value	β coefficient (95% CI)	*p*-value
**Pill burden category**														
<10 tabs/day	Reference		Reference		Reference		Reference		Reference		Reference		Reference	
10–15 tabs/day	2.39 (−2.13 to 6.91)	0.30	0.97 (−3.94 to 5.89)	0.70	−1.16 (−13.95 to 11.63)	0.86	1.37 (−2.98 to 5.71)	0.54	−0.48 (−11.65 to 10.69)	0.93	0.18 (0.08 to 0.29)	**0.001**	0.30 (−10.75 to 11.35)	0.96
>15 tabs/day	3.96 (−0.68 to 8.60)	0.09	2.41 (−2.64 to 7.46)	0.35	−10.56 (−23.69 to 2.56)	0.11	1.52 (−2.94 to 5.97)	0.50	−2.04 (−13.50 to 9.43)	0.73	0.19 (0.08 to 0.30)	**0.001**	8.02 (−3.32 to 19.36)	0.16
Age ≥65 years	−7.46 (−11.05 to −3.86)	**<0.001**	−1.39 (−5.48 to 2.70)	0.50	−1.02 (−11.74 to 9.70)	0.85	−0.92 (−4.53 to 2.69)	0.62	0.77 (−8.50 to 10.05)	0.87	−0.10 (−0.26 to −0.08)	**<0.001**	−14.33 (−23.29 to −5.36)	**0.002**
Female sex	−1.39 (−5.12 to 2.35)	0.46	−0.93 (−4.97 to 3.11)	0.65	3.98 (−6.60 to 14.55)	0.46	−0.87 (−4.44 to 2.70)	0.63	2.26 (−6.90 to 11.42)	0.63	−0.04 (−0.13 to 0.05)	0.39	−5.59 (−14.69 to 3.51)	0.23
Diabetes mellitus	−2.35 (−6.93 to 2.24)	0.31	−1.14 (−6.12 to 3.83)	0.65	−4.13 (−17.15 to 8.88)	0.53	−1.96 (−6.34 to 2.42)	0.38	−4.18 (−15.43 to 7.07)	0.46	−0.15 (−0.26 to −0.04)	**0.009**	−8.15 (−19.32 to 3.01)	0.15
Hypertension	4.12 (−1.40 to 9.64)	0.14	5.05 (−0.91 to 11.00)	0.10	15.95 (0.42 to 31.49)	**0.04**	0.44 (−4.85 to 5.74)	0.87	16.90 (3.55 to 30.24)	**0.01**	0.19 (0.06 to 0.33)	**0.005**	7.22 (−6.31 to 20.75)	0.29
Cardiovascular disease	−5.22 (−9.21 to −1.23)	**0.01**	−2.23 (−6.62 to 2.16)	0.32	−11.88 (−23.27 to −0.50)	**0.04**	−2.83 (−6.69 to 1.03)	0.15	−9.77 (−19.63 to 0.08)	0.052	−0.12 (−0.22 to −0.02)	**0.02**	−6.94 (−16.83 to 2.95)	0.17
**Dialysis modality**														
HD	Reference		Reference		Reference		Reference		Reference		Reference		Reference	
HDF	15.83 (12.60 to 19.07)	**<0.001**	16.21 (12.99 to 19.43)	**<0.001**	46.29 (38.42 to 54.16)	**<0.001**	4.36 (0.50 to 8.23)	**0.03**	44.48 (38.55 to 50.41)	**<0.001**	0.31 (0.22 to 0.40)	**<0.001**	17.97 (8.85 to 27.09)	**<0.001**
PD	9.99 (5.85 to 14.14)	**<0.001**	20.36 (16.24 to 24.49)	**<0.001**	55.59 (45.51 to 65.68)	**<0.001**	1.98 (−2.97 to 6.93)	0.43	51.79 (44.19 to 59.39)	**<0.001**	0.25 (0.13 to 0.36)	**<0.001**	32.04 (20.36 to 43.73)	**<0.001**
														
Dialysis vintage ≥5 years	−8.22 (−11.80 to −4.65)	**<0.001**	−10.52 (−14.29 to −6.76)	**<0.001**	−30.17 (−39.82 to −20.52)	**<0.001**	−4.30 (−7.86 to −0.73)	**0.02**	−32.86 (−40.58 to −25.14)	**<0.001**	−0.22 (−0.31 to −0.14)	**<0.001**	−21.02 (−29.70 to −12.34)	**<0.001**
Hemoglobin <10 g/dL	1.74 (−2.14 to 5.62)	0.38	0.13 (−4.07 to 4.34)	0.95	−10.30 (−21.19 to 0.59)	0.06	−4.56 (−8.20 to −0.92)	**0.01**	−4.37 (−13.87 to 5.13)	0.37	0.003 (−0.093 to 0.099)	0.95	4.14 (−5.33 to 13.62)	0.39
Calcium <8.5 mg/dL	5.04 (0.98 to 9.09)	**0.02**	2.44 (−2.01 to 6.89)	0.28	10.46 (−1.11 to 22.03)	0.08	−0.08 (−4.02 to 3.86)	0.97	11.20 (1.24 to 21.15)	**0.03**	0.07 (−0.03 to 0.17)	0.18	2.41 (−7.67 to 12.49)	0.64
Phosphate >4.5 mg/dL	2.39 (−1.31 to 6.08)	0.20	2.22 (−1.79 to 6.22)	0.28	8.73 (−1.70 to 19.17)	0.10	−0.69 (−4.24 to 2.85)	0.70	7.81 (−1.20 to 16.83)	0.09	0.08 (−0.02 to 0.17)	0.10	10.71 (1.79 to 19.62)	**0.02**
PTH >585 pg/mL	3.15 (−0.72 to 7.03)	0.11	2.83 (−1.37 to 7.02)	0.19	−1.99 (−13.05 to 9.07)	0.72	−0.87 (−4.48 to 2.74)	0.64	−1.36 (−10.93 to 8.21)	0.78	0.009 (−0.088 to 0.105)	0.86	−2.80 (−12.25 to 6.65)	0.56
Albumin <3.8 g/dL	−6.48 (−10.16 to −2.80)	**0.001**	−2.88 (−6.98 to 1.23)	0.17	−9.39 (−20.10 to 1.32)	0.09	−6.99 (−10.46 to −3.52)	**<0.001**	−6.19 (−15.49 to 3.11)	0.19	−0.15 (−0.24 to −0.06)	**0.002**	−15.36 (−24.37 to −6.36)	**0.001**
HbA1c ≥6.5%	−2.83 (−7.02 to 1.37)	0.19	−0.59 (−5.29 to 4.11)	0.80	1.32 (−10.87 to 13.52)	0.83	−1.68 (−5.62 to 2.27)	0.40	−1.90 (−12.52 to 8.71)	0.72	−0.12 (−0.23 to −0.02)	**0.02**	−5.18 (−15.43 to 5.07)	0.32

**Abbreviations:** BKD: Burden of Kidney Disease; CI: confidence interval; CKD-MBD: chronic kidney disease–mineral and bone disorder; EKD: Effects of Kidney Disease; EQ-5D-5L: EuroQol 5-Dimension 5-Level; EQ-VAS: EuroQol Visual Analogue Scale; HD: hemodialysis; HDF: hemodiafiltration; HRQoL: health-related quality of life; MCS-12: Mental Component Summary; PCS-12: Physical Component Summary; PD: peritoneal dialysis; PTH: parathyroid hormone; SPKD: Symptoms and Problems of Kidney Disease.

Dialysis modality was strongly associated with HRQoL outcomes in univariable analyses. Compared with HD, both HDF and PD were associated with significantly higher PCS-12, MCS-12, BKD, EKD, EQ-5D-5L index, and EQ-5D-5L-VAS scores. Dialysis vintage ≥5 years was associated with significantly lower HRQoL scores across most KDQOL-36 domains and EQ-5D-5L outcomes. Hypoalbuminemia (<3.8 g/dL) was also associated with lower PCS-12, SPKD, EQ-5D-5L index, and EQ-5D-5L-VAS scores (Table 2).

**Figure 1.**
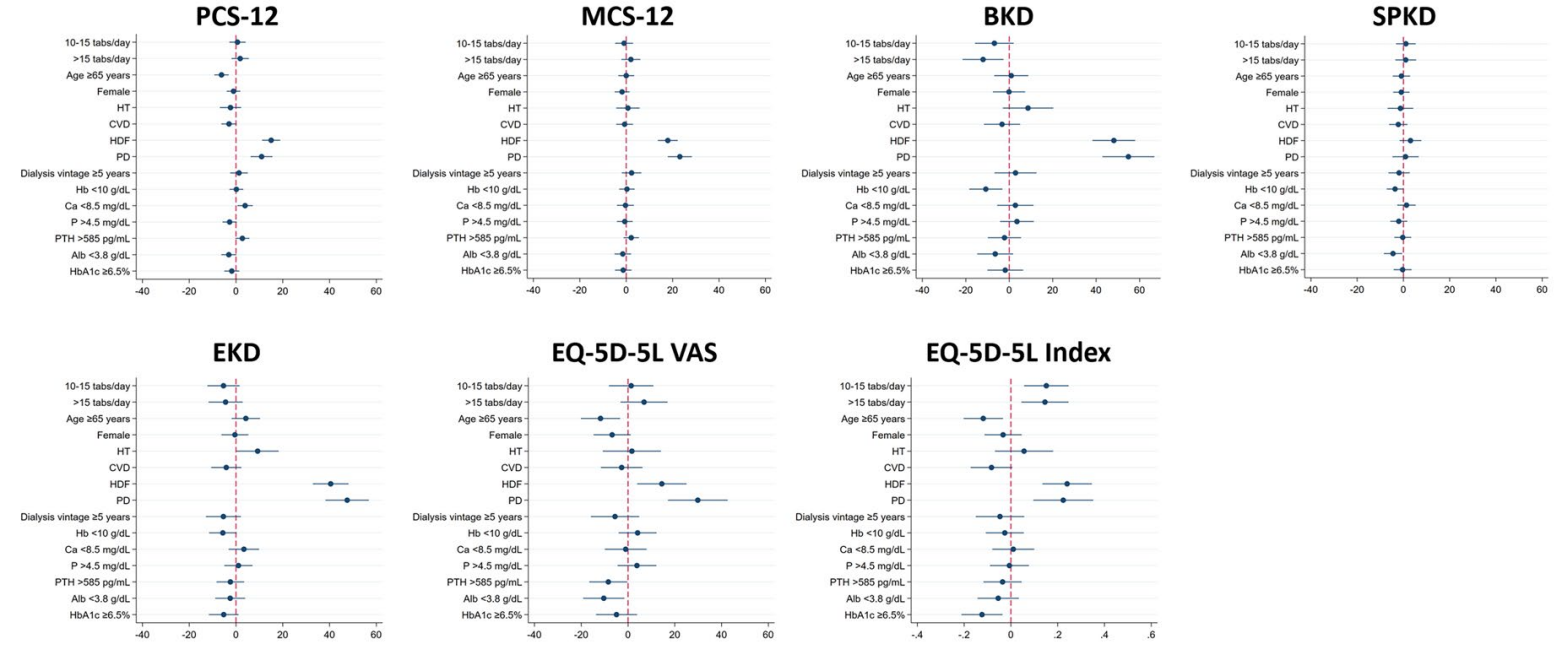
Forest plots of multivariable linear regression analyses for health-related quality-of-life outcomes in patients receiving dialysis. Panels show adjusted β coefficients and 95% confidence intervals for PCS-12, MCS-12, BKD, SPKD, EKD, EQ-5D-5L VAS, and EQ-5D-5L Index. The vertical dashed line indicates the null value (β = 0). Positive β coefficients indicate higher scores, and negative β coefficients indicate lower scores, for the listed predictor relative to its reference category. **Abbreviations:** Alb, Albumin; BKD, Burden of Kidney Disease; Ca, Calcium; EKD, Effects of Kidney Disease; EQ-5D-5L, EuroQol 5-Dimension 5-Level; Hb, Hemoglobin; HbA1c, Glycated Hemoglobin; HD, Hemodialysis; HDF, Hemodiafiltration; MCS-12, Mental Component Summary-12; P, Phosphate; PCS-12, Physical Component Summary-12; PD, Peritoneal Dialysis; PTH, Parathyroid Hormone; SPKD, Symptoms and Problems of Kidney Disease; VAS, Visual Analogue Scale.

In multivariable linear regression analyses adjusting for dialysis modality, comorbidities, dialysis vintage, and laboratory parameters, pill burden remained not significantly associated with PCS-12, MCS-12, SPKD, EKD, or EQ-5D-5L-VAS scores. However, patients receiving >15 tablets/day had significantly lower BKD scores compared with those receiving <10 tablets/day (β −12.01, 95% CI −21.42 to −2.59, *p* = 0.01). In contrast, higher pill burden remained independently associated with higher EQ-5D-5L index in both the 10–15 tablets/day group (β 0.15, 95% CI 0.05–0.24, *p* = 0.003) and the >15 tablets/day group (β 0.15, 95% CI 0.05–0.25, *p* = 0.003) (Table 3 and Figure 1).

**Table 3.** Multivariable associations between clinical Variables and HRQoL measures among patients receiving maintenance dialysis.

	PCS-12		MCS-12		BKD		SPKD		EKD		EQ-5D-5L Index		EQ-5D-5L-VAS	
Variable	β coefficient (95% CI)	*p*-value	β coefficient (95% CI)	*p*-value	β coefficient (95% CI)	*p*-value	β coefficient (95% CI)	*p*-value	β coefficient (95% CI)	*p*-value	β coefficient (95% CI)	*p*-value	β coefficient (95% CI)	*p*-value
**Pill burden category**														
<10 tabs/day	Reference		Reference		Reference		Reference		Reference		Reference		Reference	
10–15 tabs/day	0.72 (−2.77 to 4.22)	0.68	−0.72 (−4.61 to 3.16)	0.71	−6.95 (−15.87 to 1.97)	0.13	0.86 (−3.37 to 5.09)	0.69	−5.43 (−12.33 to 1.48)	0.12	0.15 (0.05 to 0.24)	**0.003**	1.13 (−8.41 to 10.66)	0.82
>15 tabs/day	1.86 (−1.83 to 5.55)	0.32	2.12 (−1.98 to 6.22)	0.31	−12.01 (−21.42 to −2.59)	**0.01**	1.17 (−3.30 to 5.63)	0.61	−4.38 (−11.66 to 2.91)	0.24	0.15 (0.05 to 0.25)	**0.003**	7.62 (−2.44 to 17.68)	0.14
Age ≥65 years	−6.20 (−9.29 to −3.12)	**<0.001**	0.00 (−3.43 to 3.43)	1.00	0.96 (−6.91 to 8.82)	0.81	−0.78 (−4.51 to 2.95)	0.68	4.23 (−1.86 to 10.31)	0.17	−0.12 (−0.20 to −0.03)	**0.007**	−11.55 (−19.96 to −3.14)	**0.007**
Female sex	−0.27 (−3.96 to 3.42)	0.88	−0.08 (−4.18 to 4.02)	0.97	−0.96 (−10.37 to 8.45)	0.84	−2.70 (−7.17 to 1.76)	0.23	−1.08 (−8.36 to 6.21)	0.77	−0.09 (−0.19 to 0.01)	0.09	−8.25 (−18.31 to 1.81)	0.11
Hypertension	−2.72 (−7.17 to 1.73)	0.23	0.15 (−4.80 to 5.10)	0.95	8.59 (−2.76 to 19.94)	0.14	−1.62 (−7.00 to 3.76)	0.55	9.08 (0.30 to 17.87)	**0.04**	0.04 (−0.08 to 0.17)	0.47	−0.69 (−12.82 to 11.45)	0.91
Cardiovascular disease	−2.79 (−6.01 to 0.43)	0.09	−0.34 (−3.92 to 3.24)	0.85	−3.32 (−11.54 to 4.89)	0.43	−2.11 (−6.00 to 1.79)	0.29	−4.09 (−10.45 to 2.26)	0.21	−0.08 (−0.17 to 0.01)	0.08	−1.61 (−10.39 to 7.17)	0.72
**Dialysis modality**														
HD	Reference		Reference		Reference		Reference		Reference		Reference		Reference	
HDF	14.92 (11.04 to 18.79)	**<0.001**	17.79 (13.48 to 22.09)	**<0.001**	48.05 (38.17 to 57.93)	**<0.001**	2.72 (−1.96 to 7.41)	0.25	40.34 (32.69 to 47.99)	**<0.001**	0.23 (0.13 to 0.34)	**<0.001**	13.16 (2.60 to 23.73)	**0.02**
PD	10.84 (6.16 to 15.53)	**<0.001**	22.96 (17.75 to 28.17)	**<0.001**	54.81 (42.86 to 66.76)	**<0.001**	0.76 (−4.91 to 6.42)	0.79	47.46 (38.21 to 56.71)	**<0.001**	0.22 (0.09 to 0.35)	**0.001**	28.97 (16.20 to 41.75)	**<0.001**
														
Dialysis vintage ≥5 years	1.14 (−2.65 to 4.93)	0.55	2.12 (−2.10 to 6.33)	0.32	2.80 (−6.87 to 12.47)	0.57	−2.21 (−6.79 to 2.38)	0.34	−5.54 (−13.03 to 1.94)	0.15	−0.06 (−0.16 to 0.05)	0.27	−7.05 (−17.39 to 3.29)	0.18
Hemoglobin <10 g/dL	0.28 (−2.69 to 3.25)	0.85	0.51 (−2.79 to 3.82)	0.76	−10.75 (−18.32 to −3.17)	**0.006**	−3.46 (−7.05 to 0.13)	0.06	−5.56 (−11.42 to 0.30)	0.06	−0.02 (−0.10 to 0.06)	0.61	4.98 (−3.12 to 13.08)	0.23
Calcium <8.5 mg/dL	3.95 (0.66 to 7.23)	**0.02**	−0.20 (−3.85 to 3.45)	0.91	2.75 (−5.63 to 11.13)	0.52	1.21 (−2.76 to 5.19)	0.55	3.35 (−3.14 to 9.83)	0.31	0.007 (−0.082 to 0.096)	0.88	−1.06 (−10.02 to 7.90)	0.82
Phosphate >4.5 mg/dL	−2.74 (−5.85 to 0.37)	0.08	−0.57 (−4.03 to 2.89)	0.75	3.39 (−4.54 to 11.32)	0.40	−2.43 (−6.19 to 1.33)	0.20	0.87 (−5.27 to 7.01)	0.78	−0.02 (−0.10 to 0.06)	0.64	2.64 (−5.84 to 11.12)	0.54
PTH >585 pg/mL	2.66 (−0.33 to 5.64)	0.08	2.03 (−1.29 to 5.35)	0.23	−2.16 (−9.78 to 5.46)	0.58	−0.21 (−3.82 to 3.40)	0.91	−2.39 (−8.28 to 3.51)	0.43	−0.03 (−0.11 to 0.05)	0.41	−8.55 (−16.70 to −0.41)	**0.04**
Albumin <3.8 g/dL	−3.28 (−6.48 to −0.08)	**0.045**	−1.79 (−5.35 to 1.77)	0.32	−6.52 (−14.69 to 1.65)	0.12	−4.81 (−8.69 to −0.94)	**0.02**	−2.62 (−8.94 to 3.70)	0.41	−0.07 (−0.15 to 0.02)	0.14	−12.04 (−20.77 to −3.31)	**0.01**
HbA1c ≥6.5%	−1.75 (−5.13 to 1.62)	0.31	−1.32 (−5.07 to 2.44)	0.49	−1.58 (−10.20 to 7.04)	0.72	0.40 (−3.69 to 4.48)	0.85	−4.96 (−11.63 to 1.71)	0.14	−0.10 (−0.19 to −0.01)	**0.03**	−2.78 (−11.99 to 6.43)	0.55

**Abbreviations:** BKD: Burden of Kidney Disease; CI: confidence interval; CKD-MBD: chronic kidney disease–mineral and bone disorder; EKD: Effects of Kidney Disease; EQ-5D-5L: EuroQol 5-Dimension 5-Level; EQ-VAS: EuroQol Visual Analogue Scale; HD: hemodialysis; HDF: hemodiafiltration; HRQoL: health-related quality of life; MCS-12: Mental Component Summary; PCS-12: Physical Component Summary; PD: peritoneal dialysis; PTH: parathyroid hormone; SPKD: Symptoms and Problems of Kidney Disease.

Among other independent predictors, age ≥65 years was associated with significantly lower PCS-12, EQ-5D-5L index, and EQ-5D-5L-VAS scores. HDF and PD modalities remained independently associated with significantly better PCS-12, MCS-12, BKD, EKD, EQ-5D-5L-VAS scores and EQ-5D-5L index compared with HD. Hypoalbuminemia (<3.8 g/dL) was independently associated with lower PCS-12, SPKD, and EQ-5D-5L-VAS scores, while elevated HbA1c ≥6.5% was associated with lower EQ-5D-5L index scores (Table 3 and Figure 1).

### Health-related quality-of-life scores according to pill burden and dialysis modality

Unadjusted HRQoL scores stratified by pill burden category and dialysis modality are shown in Supplementary Table S2. Overall, there were no significant differences in PCS-12, MCS-12, BKD, SPKD, EKD, or EQ-5D-5L-VAS scores across pill burden categories. However, the EQ-5D-5L index score differed significantly according to pill burden, with higher scores observed among patients receiving ≥10 tablets/day (overall *p* = 0.04).

Among HD patients, higher pill burden was associated with significantly higher SPKD scores (*p* = 0.046), EQ-5D-5L-VAS scores (*p* = 0.01), and EQ-5D-5L index (*p* = 0.004).

Among HDF patients, BKD and EKD scores differed significantly across pill burden categories (*p* = 0.047 and *p* = 0.02, respectively). Patients receiving 10–15 tablets/day had the highest BKD scores, whereas EKD scores tended to decrease with increasing pill burden. No significant differences were observed in PCS-12, MCS-12, SPKD, EQ-5D-5L-VAS, or EQ-5D-5L index scores.

Among PD patients, EQ-5D-5L-VAS scores differed significantly according to pill burden category (*p* = 0.04), with lower VAS scores observed in patients receiving >15 tablets/day. Other KDQOL-36 domains and EQ-5D-5L index did not significantly differ across pill burden groups.

Across dialysis modalities, PD patients generally demonstrated the highest unadjusted HRQoL scores, particularly in MCS-12, BKD, EKD, and EQ-5D-5L-VAS domains, whereas HD patients had the lowest overall EQ-5D-5L index.

## Discussion

In this cross-sectional study of maintenance dialysis patients, we evaluated the association between pill burden and HRQoL across multiple dialysis modalities using validated Thai versions of the KDQOL-36 and EQ-5D-5L instruments. The principal finding of our study was that pill burden was not consistently associated with worse HRQoL after adjustment for dialysis modality, comorbidities, dialysis vintage, and laboratory parameters. In contrast, dialysis modality and several clinical factors appeared to have a stronger association with patient-reported HRQoL outcomes.

Although polypharmacy and treatment burden have generally been reported to negatively affect quality of life in patients with ESKD [8], our findings suggest that this relationship may be more complex and demonstrate some discordance with previous studies [8]. These discrepancies may be attributable to differences in assessment tools, study populations, and statistical adjustment models. In unadjusted analyses, patients with a higher pill burden demonstrated significantly higher EQ-5D-5L index scores, and this association persisted after multivariable adjustment. Furthermore, higher pill burden was not independently associated with poorer scores across most KDQOL-36 domains, with the exception of BKD domain.

Several explanations may account for these findings. According to the baseline findings, scores for PCS and MCS domains were relatively low across all pill burden categories. This observation may suggest that patients receiving maintenance dialysis generally experience substantial impairments in physical and mental well-being regardless of medication burden. Therefore, the dialysis-dependent state itself may exert a greater influence on these domains than differences in pill burden alone. In contrast, the BKD domain specifically evaluates patients’ perceptions of the burden imposed by kidney disease on daily life. Patients with a higher medication burden may perceive the complexity of taking multiple medications as disruptive and burdensome, thereby negatively affecting BKD scores. Interestingly, the SPKD and EKD domain scores were relatively higher than PCS and MCS scores. One possible explanation is that a higher pill burden may reflect more intensive and optimized medical management, including better control of blood pressure, CKD–mineral and bone disorder, anemia, and cardiovascular risk factors. Effective pharmacologic treatment may improve symptom control and overall perceived health status despite increasing the number of medications required.

To further clarify these relationships, we performed multivariable analyses, which demonstrated that higher pill burden was independently associated only with the BKD domain and not with other aspects of HRQoL. Interestingly, when overall quality of life was assessed using the EQ-5D-5L index, a higher pill burden appeared to be associated with a signal toward better overall quality of life, in contrast to the BKD domain findings. This discrepancy may suggest that although a greater medication burden can increase patients’ perceived burden of treatment and interfere with daily life, it may simultaneously represent more comprehensive disease management and better control of symptoms and comorbid conditions, thereby contributing to improved overall health perception despite increased treatment complexity.

Moreover, pill burden alone may not adequately capture the true treatment burden experienced by patients undergoing maintenance dialysis. Multiple factors, including dialysis modality, nutritional status, transportation challenges, financial limitations, and the severity of comorbid conditions, may exert a greater influence on HRQoL than tablet count alone. This interpretation is supported by our findings, in which dialysis modality, along with several clinical and laboratory factors, demonstrated stronger and more consistent associations with HRQoL outcomes than pill burden itself.

Among dialysis modalities, patients receiving PD generally demonstrated the highest HRQoL scores, followed by those receiving HDF and conventional HD, except for the SPKD domain. These findings are consistent with previous studies reporting better quality of life among patients undergoing PD [12–14]. In addition, HDF was associated with higher HRQoL scores than conventional HD across several domains. Potential mechanisms include improved hemodynamic stability [15] and enhanced middle-molecule clearance [16], which may contribute to better physical and mental well-being and improve patients’ overall perception of quality of life. However, no significant association was observed for the SPKD domain. This finding aligns with previous meta-analyses [17] suggesting that HDF may not substantially improve symptom-related outcomes compared with conventional HD. For other HRQoL domains in meta-analysis, available evidence remains inconclusive owing to considerable heterogeneity among studies in patient characteristics, HDF delivery techniques, and HRQoL assessment approaches.

Several clinical factors were independently associated with poorer HRQoL. Older age was associated with lower physical function and lower EQ-5D-5L scores, which likely reflects frailty, sarcopenia, and reduced functional reserve in elderly dialysis patients. Hypoalbuminemia was also associated with lower HRQoL scores, particularly in physical and symptom-related domains, emphasizing the importance of nutritional and inflammatory status in patient well-being. Elevated HbA1c was independently associated with lower EQ-5D-5L index scores, suggesting that poor glycemic control may adversely affect perceived health status and symptom burden.

Although higher pill burden was not associated with poorer overall HRQoL in this study, maintenance dialysis patients still receive a substantial number of medications each day. Therefore, treatment strategies that maintain adequate disease control while avoiding unnecessary increases in pill burden should remain an important consideration in patient-centered care. Such approaches may help reduce treatment complexity and medication-related burden while preserving clinical effectiveness and patients’ overall treatment experience.

Our study has several strengths. First, we comprehensively evaluated multiple dimensions of HRQoL using both the KDQOL-36 and EQ-5D-5L instruments. The KDQOL-36 provides a kidney disease–specific assessment that captures the burden and impact of kidney disease, whereas the EQ-5D-5L reflects overall quality of life and general health status. Second, we included patients across three dialysis modalities, allowing broader assessment across different treatment settings. Third, we adjusted for important clinical and laboratory confounders to minimize potential bias. Additionally, the use of validated Thai versions of the KDQOL-36 and EQ-5D-5L enhances the reliability, validity, and cultural applicability of our findings.

Nevertheless, several limitations should be acknowledged. First, the cross-sectional design precludes causal inference between pill burden and HRQoL. Second, pill burden was quantified based on tablet counts without assessment of medication adherence, regimen complexity, dosing frequency, or patient-perceived treatment burden. Finally, this was a single-center study with unequal sample sizes across dialysis modalities, particularly the smaller PD group, which may have introduced selection bias, limited comparability among groups, and reduced the generalizability of our findings. Therefore, the results should be interpreted cautiously.

In conclusion, pill burden appears to have a complex relationship with health-related quality of life in patients undergoing maintenance dialysis. While a high pill burden (>15 tablets/day) was associated with an increased perceived burden of kidney disease, it also demonstrated a paradoxical association with a higher overall health index. Furthermore, dialysis modality remained an important determinant of HRQoL, with PD and HDF demonstrating more favorable HRQoL profiles compared with conventional HD. Clinicians should strive to achieve a balanced pharmacological approach that optimizes clinical outcomes while minimizing unnecessary treatment burden.

## Supplementary Material

Supplemental Material

## Data Availability

The data presented in this study are available on request from the corresponding author. The data are not publicly available due to privacy and ethical restrictions.
