# Supplementary material for: Pill burden and health-related quality of life across dialysis modalities: a multimodal assessment using KDQOL-36 and eQ-5D-5L
Source: Ren Fail. 2026 Aug 6;48(1):2713235. doi: 10.1080/0886022X.2026.2713235 (PMC13455696; doi:10.1080/0886022X.2026.2713235)
Supplement: Supplementary file 1 [file IRNF_A_2713235_SM7634.pdf]

**Supplementary Table S1.** Classification and pharmacologic classes of medications included in the study

| Medication category | Pharmacologic class |
|---|---|
| Antihypertensive drugs | ACE inhibitor, Angiotensin II receptor blockers, Beta-blockers, Calcium channel blockers, Centrally acting agent, Direct vasodilator |
| CKD–MBD drugs | Active vitamin D and analogues, Vitamin D supplement, Calcimimetic, Calcium-based phosphate binder, Non-calcium-based phosphate binders |
| Supplements | Iron supplement, Folic acid |
| Lipid-lowering drugs | Statins, Fibrates |
| Sodium bicarbonate | Systemic alkalinizing agent |
| Glucose-lowering drugs | DPP-4 inhibitors, GLP-1 receptor agonists, Insulin preparations, SGLT2 inhibitor, Sulfonylureas, Thiazolidinedione |
| Diuretics | Loop diuretic, Thiazide diuretic |
| Other medications | Anticoagulants, Antiplatelet agents, Uric lowering drugs, anticoagulant |

**Abbreviations:** ACE, Angiotensin-Converting Enzyme; CKD-MBD, Chronic Kidney Disease-Mineral Bone Disorder; DPP-4, Dipeptidyl Peptidase-4; GLP-1, Glucagon-Like Peptide-1; SGLT2, Sodium–Glucose Cotransporter 2.

**Supplementary Table S2.** Median Health-Related Quality-of-Life Scores According to Daily Pill Burden and Dialysis Modality. Values are presented as median (25th–75th percentile).

| **PCS-12** | | | | | |
|---|---|---|---|---|---|
| **Mode** | **<10 tabs/day** | **10–15 tabs/day** | **>15 tabs/day** | **Total** | **p-value** |
| **HD** | 22.29 (20.58–23.81) | 23.14 (20.59–27.46) | 23.82 (22.68–31.23) | 23.09 (20.64–28.83) | 0.09 |
| **HDF** | 36.75 (32.79–49.48) | 43.70 (33.37–49.89) | 42.25 (36.16–50.21) | 41.90 (33.98–50.17) | 0.57 |
| **PD** | 40.92 (34.82–45.71) | 33.75 (29.25–44.23) | 28.61 (26.77–30.82) | 36.10 (28.93–43.84) | 0.16 |
| **Overall** | 31.10 (22.29–42.99) | 32.37 (25.90–44.65) | 36.02 (23.82–45.29) | 32.78 (23.33–44.25) | 0.23 |
| **MCS-12** | | | | | |
| **Mode** | **<10 tabs/day** | **10–15 tabs/day** | **>15 tabs/day** | **Total** | **p-value** |
| **HD** | 31.54 (28.67–36.77) | 32.03 (29.07–34.62) | 32.43 (30.27–34.79) | 32.08 (28.87–36.20) | 0.92 |
| **HDF** | 48.03 (42.61–57.39) | 49.44 (39.07–56.76) | 51.13 (47.02–60.10) | 50.20 (43.04–57.75) | 0.28 |
| **PD** | 52.42 (46.25–61.15) | 55.16 (46.13–59.85) | 58.25 (53.16–64.17) | 54.88 (46.70–60.51) | 0.61 |
| **Overall** | 41.62 (31.54–53.76) | 44.66 (33.24–55.16) | 44.28 (33.21–56.78) | 43.52 (33.19–55.34) | 0.64 |
| **BKD** | | | | | |
| **Mode** | **<10 tabs/day** | **10–15 tabs/day** | **>15 tabs/day** | **Total** | **p-value** |
| **HD** | 0.00 (0.00–6.25) | 0.00 (0.00–0.00) | 6.25 (0.00–18.75) | 0.00 (0.00–12.50) | 0.20 |
| **HDF** | 68.75 (37.50–87.50) | 62.50 (25.00–81.25) | 50.00 (25.00–62.50) | 59.38 (25.00–75.00) | **0.047** |
| **PD** | 87.50 (56.25–93.75) | 62.50 (31.25–87.50) | 25.00 (18.75–56.25) | 71.88 (34.38–87.50) | 0.06 |
| **Overall** | 25.00 (0.00–78.13) | 31.25 (0.00–68.75) | 18.75 (6.25–56.25) | 25.00 (0.00–68.75) | 0.42 |
| **SPKD** | | | | | |
| **Mode** | **<10 tabs/day** | **10–15 tabs/day** | **>15 tabs/day** | **Total** | **p-value** |
| **HD** | 75.00 (70.83–83.33) | 79.17 (72.92–83.33) | 83.33 (79.17–87.50) | 79.17 (72.92–83.33) | **0.046** |
| **HDF** | 84.38 (81.25–91.67) | 85.42 (77.08–93.75) | 81.25 (75.00–89.58) | 84.38 (77.08–90.63) | 0.73 |
| **PD** | 85.42 (70.83–90.63) | 79.17 (70.83–85.42) | 87.50 (70.83–87.50) | 84.38 (70.83–87.50) | 0.86 |
| **Overall** | 82.29 (71.88–86.46) | 81.25 (75.00–85.42) | 83.33 (75.00–87.50) | 81.25 (72.92–87.50) | 0.58 |
| **EKD** | | | | | |
| **Mode** | **<10 tabs/day** | **10–15 tabs/day** | **>15 tabs/day** | **Total** | **p-value** |
| **HD** | 25.00 (21.88–37.50) | 34.38 (18.75–40.63) | 34.38 (25.00–46.88) | 31.25 (21.88–43.75) | 0.31 |
| **HDF** | 90.63 (78.13–93.75) | 78.13 (62.50–84.38) | 75.00 (65.63–84.38) | 78.13 (67.19–90.63) | **0.02** |
| **PD** | 92.19 (71.88–96.88) | 90.63 (75.00–93.75) | 90.63 (50.00–93.75) | 90.63 (73.44–95.31) | 0.58 |
| **Overall** | 59.38 (26.56–92.19) | 62.50 (34.38–81.25) | 53.13 (37.50–78.13) | 59.38 (31.25–84.38) | 0.87 |
| **EQ-5D-5L VAS** | | | | | |
| **Mode** | **<10 tabs/day** | **10–15 tabs/day** | **>15 tabs/day** | **Total** | **p-value** |
| **HD** | 20.00 (10.00–50.00) | 30.00 (20.00–50.00) | 50.00 (40.00–80.00) | 40.00 (15.00–55.00) | **0.01** |
| **HDF** | 55.00 (50.00–80.00) | 60.00 (25.00–80.00) | 65.00 (45.00–85.00) | 60.00 (47.50–80.00) | 0.89 |
| **PD** | 80.00 (75.00–82.50) | 70.00 (50.00–75.00) | 70.00 (70.00–70.00) | 75.00 (70.00–80.00) | **0.04** |
| **Overall** | 50.00 (17.50–80.00) | 50.00 (25.00–75.00) | 60.00 (40.00–80.00) | 50.00 (25.00–80.00) | 0.34 |

| EQ-5D-5L Index | | | | | |
|---|---|---|---|---|---|
| **Mode** | **<10 tabs/day** | **10–15 tabs/day** | **>15 tabs/day** | **Total** | **p-value** |
| **HD** | 0.35 (0.09–0.73) | 0.63 (0.41–0.76) | 0.75 (0.68–0.83) | 0.69 (0.34–0.81) | **0.004** |
| **HDF** | 0.83 (0.73–0.94) | 0.94 (0.88–1.00) | 0.89 (0.82–0.94) | 0.89 (0.82–0.94) | 0.13 |
| **PD** | 0.84 (0.75–0.94) | 0.79 (0.73–0.88) | 0.69 (0.63–0.76) | 0.79 (0.72–0.91) | 0.11 |
| **Overall** | 0.74 (0.33–0.88) | 0.82 (0.72–0.94) | 0.82 (0.71–0.94) | 0.80 (0.66–0.93) | **0.04** |

**Abbreviations:** BKD, Burden of Kidney Disease; EKD, Effects of Kidney Disease; EQ-5D-5L, EuroQol 5-Dimension 5-Level; EQ-5D-5L VAS, EuroQol 5-Dimension 5-Level Visual Analogue Scale; HD, hemodialysis; HDF, hemodiafiltration; MCS-12, Mental Component Summary; PCS-12, Physical Component Summary; PD, peritoneal dialysis; SPKD, Symptoms and Problems of Kidney Disease.
